# Effect of Oral Versus Nasal Breathing on Muscular Performance, Muscle Oxygenation, and Post-Exercise Recovery

**DOI:** 10.3390/sports13100368

**Published:** 2025-10-20

**Authors:** Morgan Lévénez, Clément Lévêque, Capucine Lafère, François Guerrero, Costantino Balestra, Pierre Lafère

**Affiliations:** 1 Environmental, Occupational, Aging (Integrative) Physiology Laboratory, Haute Ecole Bruxelles-Brabant (HE2B), 1160 Brussels, Belgium; morganlevenez@gmail.com (M.L.); c.leveque.research@gmail.com (C.L.); cbalestra@he2b.be (C.B.); 2 Life Science Section, Lycée Sainte-Anne, 29200 Brest, France; 3 Laboratoire ORPHY EA 4324, Univ Brest, 29200 Brest, France; francois.guerrero@univ-brest.fr; 4 DAN Europe Research Division (Roseto-Brussels), 1160 Brussels, Belgium; 5 Physical Activity Teaching Unit, Motor Sciences Department, Université Libre de Bruxelles (ULB), 1050 Brussels, Belgium; 6 Anatomical Research and Clinical Studies, Vrije Universiteit Brussels (VUB), 1050 Brussels, Belgium; 7 Department of Anesthesia, Polyclinique du Trégor, Groupe HGO, 22300 Lannion, France

**Keywords:** flow-mediated dilation, muscle, skeletal/metabolism/physiology, near-infrared spectroscopy, nitric oxide, nose versus mouth breathing, oxygen saturation, Wingate anaerobic test

## Abstract

Nitric oxide (NO) plays a crucial role in muscle oxidative capacity, which predicts muscle strength. This study aimed to investigate whether different breathing techniques (nasal or oral breathing) affect muscle performance during acute exhaustive exercise. In our study, 49 healthy individuals (24♀/25♂; age 22.8 ± 3.4 years) performed two Wingate anaerobic tests in a counterbalanced order. Although perceived exertion was significantly higher with oral breathing (Borg Scale: 9.0 ± 1.1 vs. 8.0 ± 1.3, *p* = 0.04), breathing mode did not impact power output (peak: 749 ± 290 vs. 728 ± 284 W; average: 576 ± 217 vs. 575 ± 216 W, *p* = 0.2). NIRS data indicated no significant differences in muscle desaturation between the two breathing modes; however, nasal breathing resulted in significantly faster (0.45 ± 0.4 vs. 0.23 ± 0.12%/s, *p* = 0.02) and greater (75.2 ± 4.0 vs. 73.1 ± 3.6%, *p* = 0.04) post-exercise muscle recovery. As an indirect marker of NO bioavailability, flow-mediated dilation (FMD) was associated with a significant improvement (Pre: 107.4 ± 3.0% vs. Post: 110.3 ± 3.6%, *p* < 0.001) via nasal breathing only, with a significant difference between the two breathing modes (*p* < 0.0001). Therefore, we suggest that the nitrate–nitrite–NO pathway enhances muscle energy and function, which highlights the importance of nasal breathing.

## 1. Introduction

During physical activity, muscle performance depends on numerous factors [1,2], for example, muscle composition (fiber type, size and length, architecture, fascicle pennation angle, etc.), muscular adipose tissue, metabolic supply, insulin sensitivity, neuromuscular activation, and muscle oxidative capacity. In a recent review [1], only muscle oxidative capacity was found to be a predictor of muscle strength. Indeed, it has been demonstrated that skeletal muscle bioenergetics is a major determinant of functional mobility, defined by walking speed. The more demanding the walking task, the more significant the direct role of bioenergetics [3]. This has crucial clinical implications. Indeed, aging has been associated with a decline in skeletal muscle mitochondrial oxidative capacity, something that negatively affects one’s ability to walk [4], an essential ability for daily activities and for older adults to maintain their independence [5]. Therefore, interventions aimed at improving and maintaining muscle strength through muscle mitochondrial function—mainly, if not exclusively, physical exercise—are mandatory to reduce, postpone, or prevent mobility loss [6,7].

Among the different pathways that underpin skeletal muscle metabolism, nitric oxide (NO) has a functionally relevant role [8]. Indeed, it regulates mitochondrial respiration and biogenesis and can inhibit the electron transport that regulates oxygen consumption and ATP generation. At the same time, it can induce an increase in reactive oxygen and nitrogen species [9,10]. NO is also a major contributor to exercise hyperemia, a coping mechanism to meet increased demand for oxygen and energy substrates [11].

NO cannot be stored inside cells; therefore, it requires multiple controlling mechanisms to regulate its functions. In muscular cells, two main sources of NO exist: the nitric oxide synthase (NOS)-dependent pathway and the NOS-independent pathway. The latter is where nitrate (NO_3_) is reduced to nitrite (NO_2_) and further reduced to NO [12]; that being said, the main source of NO is the NOS-dependent pathway, which accounts for about two-thirds of NO production [13]. It involves a series of reactions catalyzed by NOS enzymes that oxidize L-arginine to NO, which requires the presence of many cofactors, including oxygen [11]. Although all NOS isoforms—neuronal NOS (nNOS), endothelial NOS (eNOS), and inducible NOS (iNOS)—are found in muscular cells, in normal conditions, iNOS is hardly detectable and is mostly expressed in response to inflammatory insult and/or oxidative stress [14]. It is also important to note that there is no compensatory relationship between NOS isoforms; if one of them is lacking, this does not trigger an increased protein expression of the other forms—this is the case in animal studies, at least [15]. Although several mechanisms are involved in NO production, including shear stress, cardiac output, and arteriovenous difference, this study focused on the potential to increase NO production through iNOS stimulation in the nasal fossae and sinus mucosae. Indeed, nasal breathing promotes NO concentration up to ten times more than what has been observed in the bronchi [16,17]; meanwhile, oral breathing can significantly reduce circulating NO, which might promote vascular dysfunction [18]. Although the evidence for this is quite old, more recent data have also suggested that nasal breathing might significantly improve the maintenance of NO production and bioavailability in order to reduce the occurrence of vascular dysfunction while exposed to extreme environments [19].

Therefore, the objective of this exploratory study was to investigate the effects of nasal breathing on muscle performance during exhaustive exercise. Nasal breathing was hypothesized to enhance vessel diameter and blood flow during exercise and, consequently, muscle oxygenation. This might be relevant for strenuous exercise routines like kendo (Japanese fencing), which has been known to increase oxidative stress and cause cellular damage [20,21].

## 2. Materials and Methods

Experimental procedures were conducted in accordance with the Declaration of Helsinki and were approved by the Academic Ethical Committee of Brussels (Belgium) (Ethic committee B 200-2022-038).

### 2.1. Experimental Protocol

After we received written informed consent and medical clearance, 49 healthy nonsmoking individuals (24 females and 25 males) volunteered for this study. They were selected from a larger population of students in physical and sports education (aged between 18 and 31 years); however, none of the participants trained in sports at a professional or elite level. They were not classified as being at risk of cardiovascular diseases according to the current Belgian recommendations on sports cardiology and exercise [22,23]. They were also instructed not to drink any alcoholic [24] or caffeinated beverages [25,26], smoke [27], or perform any kind of exercise [28,29] for 8 h prior to the experiment. These are all confounding factors of flow-mediated dilation (FMD) analysis.

Each participant, already familiarized with the procedure, completed two Wingate anaerobic tests (WAnTs) on an assault bike (Rogue Echo Bike V3.0, Rogue Fitness, Columbus, OH, USA), spaced two days apart. This air-resistance bike facilitated simultaneous engagement of both the upper and lower extremities. The test procedure we followed is well described in the literature [30]. In short, it included a warm-up of 5 min—pedaling at 50–70 rpm (approximately 50–70 watts). After warming up, the subjects rested in a sitting position on the bike for five minutes. Then, at the count of “3, 2, 1…Go!” the participants started to pedal as hard as possible for thirty seconds. Their task was to maintain the maximum cadence until the end of the test. Therefore, they were continuously motivated by verbal encouragement. Although durations such as 15, 20, or even 45 and 60 s have been described in the literature, we opted for the 30-s duration, which is the typical duration of the WAnT [31]. Afterwards, a 5 min recovery ride at a warm-up pace was carried out to help cool participants down.

Power output was recorded every 5 s. Each subject’s peak power output was defined as their highest value, and the average peak power output was calculated over the 30-s WAnT. These values were used to determine mean peak power output (PPO) and average peak power output (aPPO) across all 49 subjects for further analysis.

All procedures were performed in the Physiology Laboratory at the Haute Ecole Bruxelles Brabant. The WAnTs were performed under one of two conditions, either nasal (N) or oral breathing (O), performed in counterbalanced order. As each participant regularly engaged in sports, it is reasonable to assume they possessed enhanced respiratory control. Therefore, under the N condition, participants had athletic tape placed over their mouths to prevent any oral breathing, while for the O condition, a standard laboratory nose clip was used to prevent nasal breathing. These were placed immediately following the warm-up, approximately 20 s prior to the start of the WAnT, and removed when the recovery period began. Participants were randomly assigned to begin with either the N or O condition and vice versa for their second test. Also, the time of day for testing was kept consistent for all subjects during the second test [32], making each participant his or her own control.

### 2.2. Oxygenation: Pulse Oximetry (SPO_2_) and Near-Infrared Spectroscopy (NIRS)

Arterial oxygen saturation and vastus lateralis (VL) muscle tissue oxygenation were assessed by pulse oximetry (SPO_2_) and near-infrared spectroscopy (NIRS), respectively, as a reflection of macro- and microcirculation.

To avoid artifacts in SPO_2_ readings using a probe (Nellcor PM10N, Medtronic Canada, Brampton, ON, Canada), the probe’s placement area (second or third finger on the non-dominant hand) was cleaned with a cotton swab soaked in alcohol, and the probe was secured with hypoallergenic skin tape. The participants were also asked, as often as needed, not to squeeze this hand on the bike handle. After a minute of auto-calibration, the oximetry recording started and was maintained throughout the testing sessions, including the warm-up and recovery periods.

Muscle oxygenation of the VL was measured using a continuous-wave NIRS device (Train.Red Plus, Gelderland, The Netherlands). The probe was placed on the subject’s non-dominant leg, perpendicular to the estimated longitudinal axis of the VL, 15 cm above the superior border of the patella and 5 cm lateral to the midline of the thigh. The distance of the probe to the patella was outlined with a marker and measured to ensure consistent placement between sessions. It was attached to the skin with an adhesive patch that prevents interference from ambient light and ensures data quality; it was then secured around the thigh using a black elasticated strap. After each WAnT, we checked for a slightly depressed cutaneous area at the location of the probe to ensure that the proper contact between the probe and the skin was maintained. Detailed descriptions of the technology behind NIRS can be found in other studies that measure the light attenuation at wavelengths of 770 and 830 nm using algorithms based on a modified Beer–Lambert law [33,34]. The NIRS sampling rate was set at 1 Hz, and the device calculated the total saturation index (TSI), which is a measure of local oxygenation. All data were collected on a smart device and then transferred to a computer. The baseline TSI was determined to be the average of 30 s preceding the WAnT. The area under the curve over 30 s of exercise to exhaustion (AUC 30sec) and the difference between the baseline and the lowest TSI value (∆TSI) during the WAnT were utilized as a measure of the magnitude of oxygen desaturation. The downslope of the TSI calculated over the 30 sec of the WAnT was a measure of oxygen (O_2_) desaturation rate, while the upslope of the TSI signal was a measure of the O_2_ resaturation rate, calculated over a 10 s window immediately following the conclusion of the WAnT.

### 2.3. Flow-Mediated Dilation (FMD)

Flow-mediated dilation (FMD) is an established measure of the endothelium-dependent vasodilation mediated by nitric oxide (NO) [35]. Therefore, using a digital diagnostic ultrasound system (DP-30, Mindray, Echomedic, Ghent, Belgium), we assessed the effect of oral versus nasal breathing on main conduit arteries. We opted for the reactive hyperemia technique, whereby brachial artery diameter is measured before and 1 min after a 5 min ischemia induced by inflating a cuff placed on the forearm, as previously described [19,36,37]. The artery diameter was measured manually with an electronic caliper (provided by the ultrasonography software) in a three-fold repetition pattern. The average diameter over these three measurements was used to calculate the percent increase in arterial diameter from the resting state to maximal dilation. All measurements were performed by experienced operators who conduct more than 100 scans/year, which is a recommended criterion to maintain competency in the FMD method [38].

### 2.4. Statistical Analysis

The sample size was established a priori using G*Power 3.1 software (v.3.1.9.6, Heinrich-Heine-Universität Düsseldorf, Düsseldorf, Germany), and the expected effect size was set at 0.5, the α level was set at 0.05, and the power (1-β) was set at 0.95, leading to a sample size of 47 individuals.

The normality of the results was verified using the Kolmogorov–Smirnov test. Depending on the distribution, comparisons between oral versus nasal breathing were performed using either a paired *t*-test or a Wilcoxon signed-rank test; meanwhile, for comparisons between men and women, we used their equivalent for unpaired data, either an unpaired *t*-test or a Mann–Whitney U test, respectively. Finally, comparisons between results at different times and the baseline were carried out using repeated-measure one-way ANOVA tests or a Friedman test.

All statistical tests were performed using a standard computer statistical package, GraphPad Prism version 10.4.1 for Mac (GraphPad Software, San Diego, CA, USA). A threshold of *p* < 0.05 was considered statistically significant. All data are presented as means ± standard deviation (SD).

## 3. Results

The baseline demographics of the participants (24♀/25♂) are summarized in Table 1. Men were significantly taller and heavier than women, but their body compositions were similar.

### 3.1. Power Output

**Figure 1:**
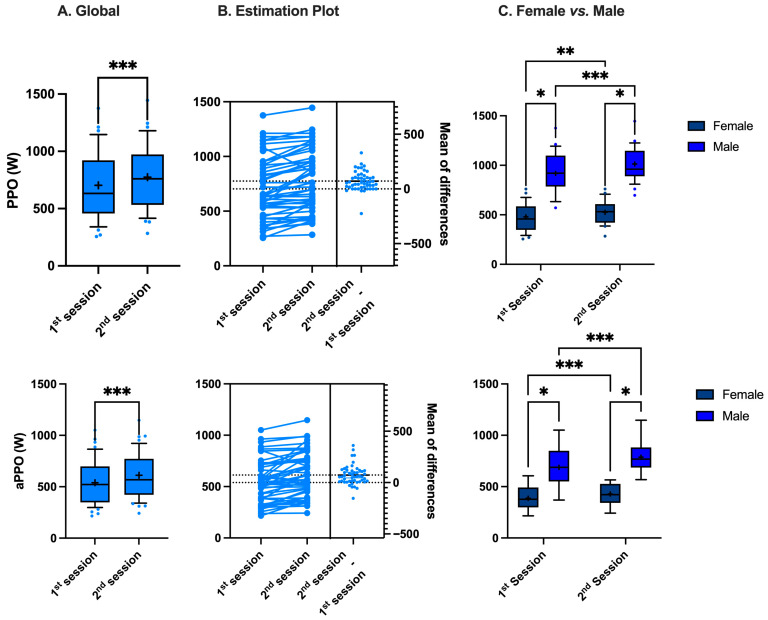
Difference in peak power output (PPO) and average peak power output (aPPO) between sessions (**A**,**B**) and between female and male (**C**) (*: *p* < 0.05, **: *p* < 0.01, ***: *p* < 0.001; (**A**): paired *t*-test, (**C**): Mann–Whitney).

A significant increase in PPO and aPPO was observed between the first and second sessions, from 703 ± 283 to 775 ± 287 W (*p* < 0.001, paired *t*-test) and from 539 ± 211 to 612 ± 216 W (*p* < 0.001, paired *t*-test), respectively (Figure 1A). This represents an increase of 9.9 ± 11.9% and 15.1 ± 19.2%, respectively. There is also a significant difference in both PPO and aPPO between females and males (Figure 1C) (♀: 8.5 ± 2.9 and 6.9 ± 2.2 W/kg; ♂: 13.2 ± 3.5 and 10.0 ± 2.8 W/kg, respectively, *p* < 0.001, Mann–Whitney). However, this gender difference remains constant across sessions (PPO: 0.8 ± 1.0 (♀) vs. 1.0 ± 1.1 (♂) W/kg, *p* = 0.06 Mann–Whitney; aPPO: 0.8 ± 1.0 (♀) vs. 1.3 ± 1.6 (♂) W/kg, *p* = 0.4, Mann–Whitney). When transformed as a percentage variation to evaluate the magnitude of the change, the increase in both PPO and aPPO is similar in females and males (PPO: 9.5 ± 12.5 (♀) vs. 10.2 ± 11.6 (♂) %; aPPO: 13.2 ± 16.0 (♀) vs. 17.0 ± 22.0 (♂) %, *p* = 0.3 and *p* = 0.68, respectively, Mann–Whitney).

**Figure 2:**
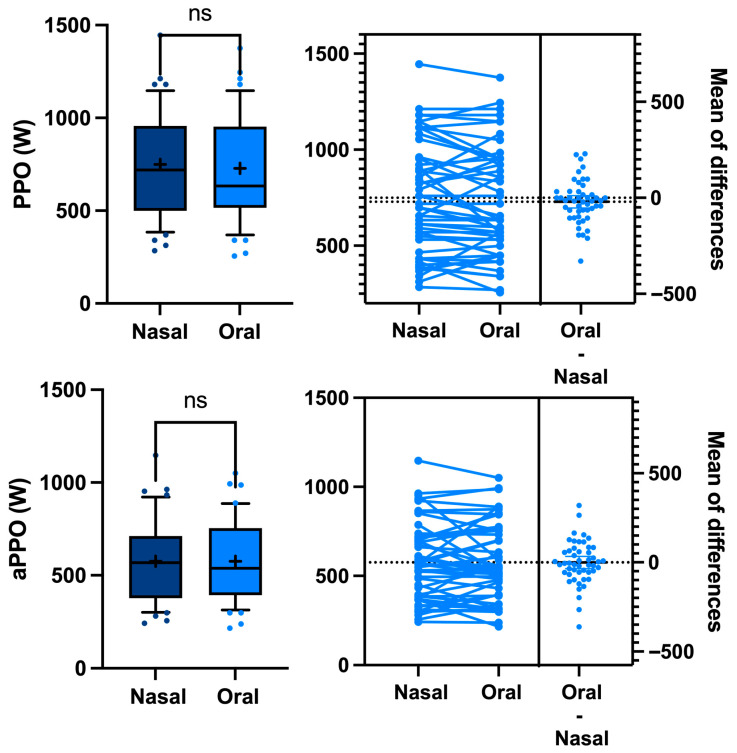
Difference in peak power output (PPO) and average peak power output (aPPO) between oral vs. nasal breathing during a Wingate anaerobic test. Every participant acts as his or her own control (ns: not significant, paired *t*-test).

Although there are some gender differences and between-sessions differences, our counterbalanced crossover study design allows for a direct comparison of oral vs. nasal breathing by grouping all the results into a single analysis, showing that the respiratory mode has no effect on either the PPO or the aPPO (749 ± 290 vs. 728 ± 284 W, *p* = 0.2 paired *t*-test and 576 ± 217 vs. 575 ± 216 W, *p* = 0.2 paired *t*-test, respectively—Figure 2), which is consistent with the previous literature [39,40].

### 3.2. Oxygenation

SPO_2_ was not significantly modified across the WAnT procedure (oral: 97.9 ± 0.9 (Pre) vs. 97.6 ± 1.0 (Nadir) vs. 98.0 ± 0.9 (Post) %, *p* = 0.12 Friedman; nasal: 98.2 ± 0.7 (Pre) vs. 97.0 ± 0.9 (Nadir) vs. 98.1 ± 0.9 (Post) %, *p* = 0.07 Friedman test). Also, breathing pattern did not significantly modify SPO_2_ (Pre: *p* = 0.15; Nadir: *p* = 0.08; Post: *p* = 0.31, Wilcoxon).

**Figure 3:**
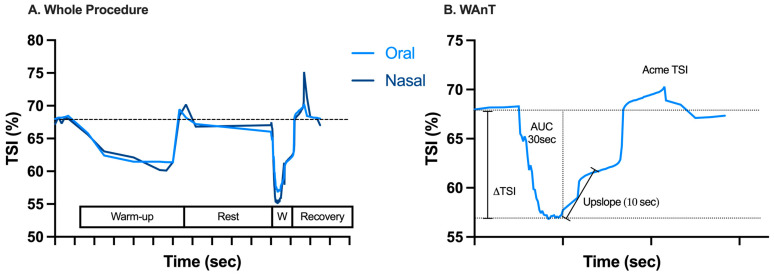
Average time plot of near-infrared spectroscopy-derived tissue oxygen saturation (TSI) signal after elimination of artifacts measured across the 49 subjects through (**A**) the whole procedure and (**B**) the WAnT representative of oral breathing, as well as the variables assessed (AUC = area under the curve; ΔTSI = the difference between baseline TSI and the lowest TSI value). On the X-axis, each gradation corresponds to 60 s.

The average global TSI signal profile across the 49 subjects, the variables assessed, and the average of the TSI signal during WAnT are all shown in Figure 3.

Table 2 shows NIRS parameter data between conditions (oral vs. nasal breathing). No significant differences were found in the desaturation rate and the magnitude of this desaturation. On the other hand, when it comes to resaturation of the VL muscle, nasal breathing was responsible for a significantly faster and greater compensation than oral breathing.

### 3.3. Flow-Mediated Dilation (FMD)

**Figure 4:**
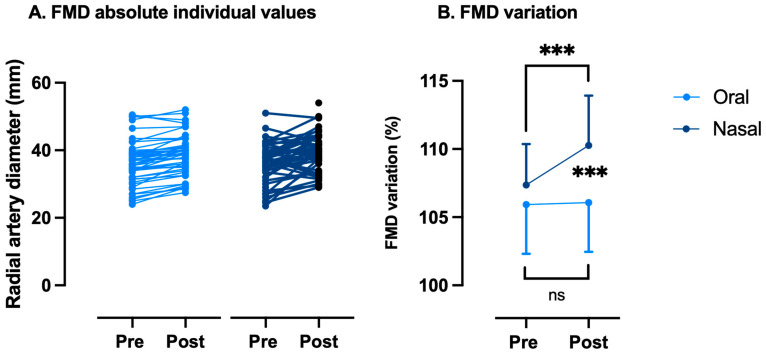
Variations in flow-mediated dilation (FMD) between oral and nasal breathing after a Wingate anaerobic test. FMD changes are expressed as follows: (**A**) absolute individual values and (**B**) % of pre-exercise values. Every participant acts as his or her own control (ns: not significant, ***: *p* < 0.001; intragroup: paired *t*-Test; intergroup: Wilcoxon).

As said in the Introduction, nasal breathing seems to be a potent physiological strategy to increase circulating NO [19,41,42], potentially modulating the effects of exhaustive exercise on FMD. Accordingly, FMD variation values were not significantly modified while breathing through the mouth (Pre: 105.9 ± 3.6% vs. Post: 106.1 ± 3.6%, *p* = 0.81 paired *t*-test). On the other hand, nasal breathing is associated with a significant improvement in FMD (Pre: 107.4 ± 3.0% vs. Post: 110.3 ± 3.6%, *p* < 0.001 paired *t*-test). The difference between the two conditions is very significant (*p* < 0.0001, Wilcoxon) (Figure 4). FMD returned to normal values 2 h after the WAnT was executed while breathing through the nose at 101.6 ± 2.5%.

## 4. Discussion

The primary finding from this study was that exercise performance improved in the second session and was significantly higher in men than women, with men’s PPO and aPPO exceeding women’s by 36% and 31%, respectively. This gender gap is larger than reported in previous studies (10–20% [43,44]), possibly due to differences in body composition [45] or exercise modality. Indeed, gender differences in power output may be smaller when adjusted for fat-free mass rather than body mass [43]. Also, the use of an assault bike, which increased the involvement of the upper body, likely widened the gap [46]. Furthermore, the influence of external factors such as verbal encouragement, motivation, and the presence of an audience cannot be overlooked [47,48]. Indeed, the fluctuation in the WAnT test–retest variability was estimated to range from 0 to as high as 22%, with a global trend toward improvement [49], which calls into question the objective intragroup and intra-subject comparisons. However, the crossover design, with participants as their own controls and random allocations for breathing modes, should have mitigated any bias and improved reliability. Indeed, our results align with previous studies [50].

PPO results fall within the 50th to 75th percentile of the Wingate muscular power test [51]. This is relevant to participants involved in club or recreational sports who consistently exercise at moderate (3.0–5.9 METs) or strenuous (6 + METs) intensities, which aligns with our sample criteria. Indeed, the participants were sports practitioners, although not at an elite level. Nonetheless, it is reasonable to assume they have enhanced experience in respiratory control. This might have skewed our results. However, the counterbalanced crossover design over two days, as well as devices used to prevent mixed breathing, likely minimized this issue. Moreover, although nasal breathing enhances ventilatory efficiency [52], the mode of breathing did not have a significant impact on performance metrics during a high-intensity anaerobic test. This finding is corroborated by previous studies [53].

To further discuss our results, we must acknowledge some limitations. Indeed, NIRS recordings were performed on the lower limbs, while SPO_2_ and FMD were measured on upper limbs. We chose the quadriceps due to their higher oxygen consumption during cycling [54] and used the brachial artery for FMD because of operator expertise. While a positive correlation was observed between NIRS-derived oxygen resaturation rate and percent change in FMD for both the lower limbs (tibialis anterior muscle/popliteal artery) [55] and upper limbs (flexor digitorum superficialis muscle/brachial artery) [56], studies comparing both limbs are lacking. However, establishing a correlation between FMD and NIRS was not the objective of this study. In the present setting, FMD was employed as an indirect method to evaluate NO bioavailability.

Indeed, numerous studies have examined the effects of various NO-related supplements—including inorganic nitrate, nitrite, L-arginine, and L-citrulline—on exercise performance. These dietary supplements have been linked to improved performance, reduced oxygen consumption during exercise, enhanced vessel diameter and blood flow, and delayed onset of fatigue in highly active individuals [57]. Nasal breathing serves a similar function. In the context of a non-provocative 20-min dive to 10 m, whether using a half-mask (which bypasses the nose) or a full-face mask (allowing for nasal breathing only), it has been demonstrated that such conditions significantly enhance nitric oxide (NO) production [19]. Our results show enhanced vasodilation but no improved performance, which partially supports previous research on the NO hypothesis. This lack of performance improvement could be due to NO-related supplementation being primarily effective in untrained and older individuals [58], which do not match our sample criteria.

TSI also supports the involvement of NO. Previous studies have demonstrated the following: (1) a notable correlation between peak lactate levels and mean power in the WAnT, indicating that glycolytic metabolism is primarily activated, and (2) a significant increase of 78% in reactive oxygen species (ROS) free radical scavenging activities compared to pre-exercise values [59]. This suggests an increase in ROS production, as various proteins involved in glycolysis, mitochondrial electron transport, β-oxidation, and the tricarboxylic acid cycle can generate superoxide, hydrogen peroxide, and other ROS [60]. This high-intensity exercise-related oxidative stress caused by excessive ROS production may be counteracted by several NO-related mechanisms, whose bioavailability increases with nasal breathing [19]. Firstly, NO can react with superoxide to form peroxynitrite. While this nitrosative stress may be potentially damaging, it effectively removes superoxide from circulation, thereby mitigating its harmful impact on endothelial cells [61]. Secondly, NO triggers protective cellular signaling through soluble guanylate cyclase, thereby producing cGMP. This mediator activates protein kinases, safeguarding cells from oxidative damage and promoting vasodilation [62]. This process improves oxygen delivery while also accelerating the removal of metabolic by-products such as lactate, thereby reducing further oxidative stress [63]. Finally, NO regulates mitochondria, which affects both their function and cellular activity, by inhibiting respiratory chain complexes through competition with oxygen or modifying mitochondrial components, ultimately reducing oxidative stress [10]. Hypothetically—since we measured neither NO nor ROS—these mechanisms, either alone or together, might account for our results, mainly a significant increase in the resaturation rate and a stronger compensatory response when breathing through the nose. While the complete spectrum of processes involving NO is still debated, it is clear that NO interactions within metabolic networks are intricate and influenced by various factors, particularly the concentration of NO at the site of action [10].

## 5. Conclusions

The available data on oral-only or nasal-only breathing during anaerobic exercise is limited. However, we found that breathing mode did not impact power output or performance in high-intensity anaerobic exercise tests. Despite the advantages and disadvantages of each breathing mode, participant preference should not be the deciding factor [53]. Indeed, our findings also show that the nitrate–nitrite–NO pathway might enhance muscle energy and function, which highlights the importance of nasal breathing. Therefore, we call for further research into the effectiveness of nasal breathing during exercise. This research should focus on optimizing NO bioavailability for muscle recovery in both athletes and the elderly.

## Figures and Tables

**Table 1:** Demographic variables of the study participants (***: *p* < 0.001; Mann–Whitney).

	♀	♂	*p*
Age (Year)	22.3 ± 2.8	23.4 ± 4.0	0.72
Height (cm) ***	165.7 ± 5.3	179.4 ± 6.1	<0.001
Weight (kg) ***	60.4 ± 7.2	75.2 ± 12.1	<0.001
BMI (kg/m^2^)	22.0 ± 2.6	23.3 ± 3.0	0.18
Physical activity (hours/week)	3.5 ± 3.4	3.4 ± 2.3	0.80

**Table 2:** Tissue oxygen saturation (TSI) during a Wingate test while breathing either through the mouth or the nose (*: *p* < 0.05).

Variables	Oral Breathing	Nasal Breathing	*p*
AUC 30 s	274 ± 146	254 ± 124	0.55
∆TSI (%)	13.6 ± 6.52	14.4 ± 5.2	0.48
Desaturation rate (%/sec)	−0.31 ± 0.23	−0.37 ± 0.19	0.34
Resaturation rate (%/sec)	0.23 ± 0.12	0.45 ± 0.4	0.02 *
TSI max (%)	73.1 ± 3.6	75.2 ± 4.0	0.04 *

## Data Availability

The data presented in this study are available on request from the corresponding author due to restriction related to data protection of government employee.

## Abbreviations

The following abbreviations are used in this manuscript:

aPPO	Average peak power output
AUC30	Area under the curve over 30 s
FMD	Flow-mediated dilation
NIRS	Near-infrared spectroscopy
NO	Nitric oxide
NO_2_	Nitrite
NO_3_	Nitrate
NOS	Nitric oxide synthase and isoforms: neural nNOS, endothelial eNOS, and inducible iNOS
O_2_	Oxygen
PPO	Peak power output
SPO_2_	Pulse oximetry
TSI	Total saturation index
VL	Vastus lateralis muscle
WAnT	Wingate anaerobic test
